# Supplementary figures and images for: Isolation and characterization of a motility-defective mutant of Euglena gracilis
Source: PeerJ. 2020 Sep 28;8:e10002. doi: 10.7717/peerj.10002 (PMC7528813; doi:10.7717/peerj.10002)

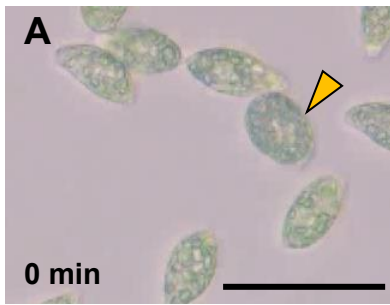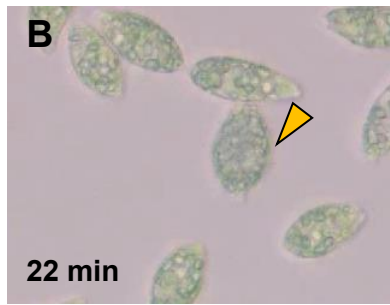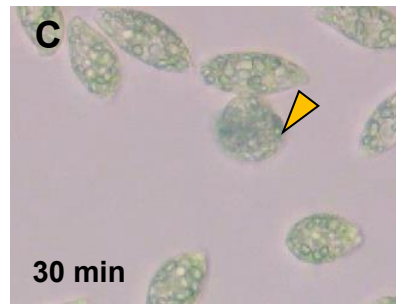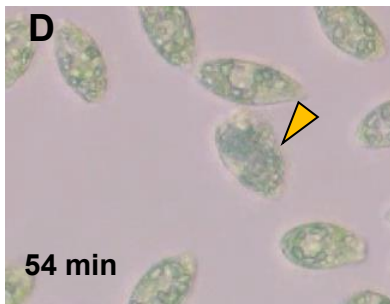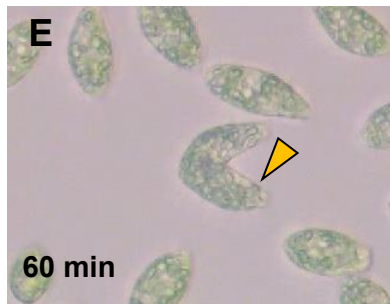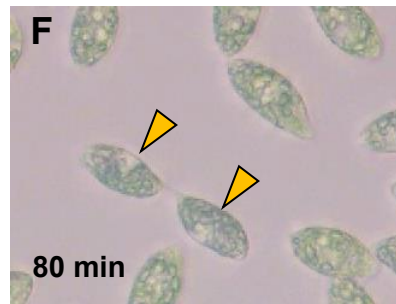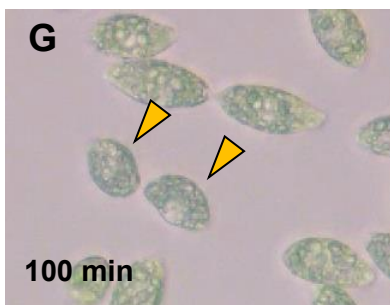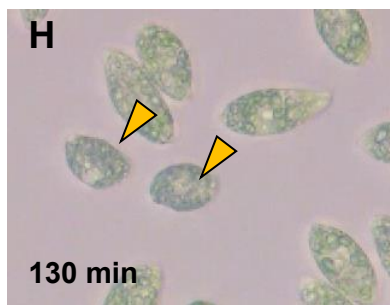

Supplement: Figure S1 — Time-lapse images of E. gracilis M-3ZFeL strain were photographed for 4 h at 1 min intervals. Ten images were extracted and cropped to highlight the status of a dividing cell. The time shown in the left bottom of each panel indicates the lapse time. Yellow triangles indicate the cell in division. Scale bar indicates 50 µm. [file peerj-08-10002-s001.pdf]
